# Examining the Role of Mental Health and Clinical Issues within Talent Development

**DOI:** 10.3389/fpsyg.2015.02042

**Published:** 2016-01-11

**Authors:** Andy Hill, Áine MacNamara, Dave Collins, Sheelagh Rodgers

**Affiliations:** ^1^Institute of Coaching and Performance, University of Central LancashirePreston, UK; ^2^Clinical Psychologist, Private PracticeInverness, UK

**Keywords:** mental health, clinical psychology, talent development, behavioral indicators, behavioral change, assessment, warning signs

## Abstract

Although significant research supports the association between physical activity and mental wellbeing, current literature acknowledges that athletes are no less susceptible to mental illness than the general population. Despite welcomed initiatives aimed at improving mental health within elite sport, these programs often fail to target young athletes; an important concern given that the genesis of many mental illnesses are recognized to occur during this critical period. Given the importance of early intervention and effective treatment, and the potentially devastating consequences of clinical issues going undiagnosed, the implications for talent identification and development (TID) become obvious. With this in mind, this study sought to examine the range of mental health issues that may impact upon developing athletes and potential consequences for the development process, specific risk and protective factors associated with talent development, along with an examination of current practices concerning the identification of mental health issues in such environments. Qualitative interviews were conducted with purposively sampled clinicians (*n* = 8) experienced in working with adolescents and/or young athletes. Inductive content analysis was undertaken, identifying four main themes: key behavioral indicators; associated risk factors; associated protective factors; and issues around identification and diagnosis. Key behavioral indicators included behavioral change, along with behaviors associated with eating disorders, anxiety and depression. Risk factors centered on family background, the performance environment, and issues surrounding adolescence. Protective factors were primarily social in nature. Finally, a lack of awareness and understanding of clinical issues, multiple causes of symptoms, non-disclosure and the need for triangulation of assessment were identified. The need for improved identification and intervention strategies was apparent, with coaches identified as well placed to detect general “warning signs” such as behavioral change. Short of integrating trained clinicians into talent development environments, as part of a triangulation process, ecologically validated assessment tools—coupled with appropriate training and signposting—could offer a practical way of flagging potential issues in developing athletes. The need for the development of such an instrument is therefore apparent. Finally, education around the influential role of family is also recommended in order to promote the protective elements and mitigate risk factors.

## Introduction

Mental health issues within elite sport have received significant media attention of late, with athletes such as Marcus Trescothick, Clarke Carlisle, and Dame Kelly Homes all offering high-profile examples of its prevalence. Although there is a significant body of literature supporting the association between physical activity and mental health (e.g., Morgan et al., 2013), as well as the erroneous assumption that mental toughness may offer a protective factor (cf. Mazzer and Rickwood, 2015), current literature acknowledges that athletes are no less susceptible to mental illness than the general populations (Markser, 2011; Bär and Markser, 2013). Indeed, recent reporting of high profile athletes discussing their mental health issues has led to a series of initiatives designed to make an impact in performance sport, such as the work of the organization State of Mind, and the Performance Matters program by the mental health charity Mind. These initiatives include education around behavioral indicators of potential clinical issues and improved signposting of referral programs within professional organizations, all aimed at providing elite athletes with appropriate support.

Despite these initiatives within sport being both necessary and welcome, there appears to be an underlying limitation to their effectiveness. Given that membership of professional bodies and high performance support usually requires athletes to have attained professional status or have been awarded a position on a performance program, what happens if the athlete requires support prior to this point? Within the UK, approximately 10% of children aged between 5 and 15 have a clinically diagnosable (i.e., can be categorized according to the Diagnostic and Statistical Manual of Mental Disorders, DSM-V; American Psychiatric Association, 2013) mental disorder (Green et al., 2004), with half of all lifetime cases of mental illness beginning by the age of 14, and three-quarters by the age of 24 (Kessler et al., 2005). Given the importance of early intervention and effective treatment (Burns and Birrell, 2014), the age groups concerned, and the potentially devastating consequences of clinical issues going undiagnosed, the implications for talent identification and development (TID) become obvious. Despite these concerns, however, there appears to be a dearth of research examining both the nature and impact of mental health issues in a TID setting and, more specifically, a dearth of research involving genuine clinical expertise. For effective understanding and expert advice on this highly sensitive issue, this is of paramount importance.

In their investigation into psycho-behavioral features of effective talent development, Hill et al. (2015) reported a range of mental health issues identified by coaches within elite rugby union academies as negatively impacting upon the athlete and potentially leading to talent derailment. These included anxiety and depression, eating disorders, perfectionistic behaviors and suicidal thoughts and feelings. Along with the obvious primary issue of detrimental impact on an athlete's wellbeing, there are also several negative implications for the talent development process itself. For example, research has shown that symptoms of anxiety and depression can predict avoidance-based coping behaviors within students (Grant et al., 2013). Avoidance coping, defined as an attempt to “minimize, deny, or otherwise circumvent managing specific stressors” (Grant et al., 2013), is particularly detrimental to development, as it mitigates the interaction between the athlete and developmental challenge (Phillips et al., 2010; Collins and MacNamara, 2012). In possible contrast, developmental challenge, though described as inherently stressful, has been shown to be a key driver of development by enabling young performers to develop and refine the psycho-behavioral skills (e.g., resilience) required to negotiate their pathway to excellence (Collins and MacNamara, 2012; Sarkar and Fletcher, 2014). Grant et al. (2013) also demonstrated that the relationship between avoidance coping and anxiety and depression was reciprocal, suggesting that if an athlete choses to consistently employ avoidance-coping behaviors to mediate developmental challenge, then they are at an increased risk of developing depressive or anxious symptoms, which may in turn lead to a vicious cycle of avoidance and anxiety and/or depression.

High levels of perfectionism, recognized as prevalent yet potentially maladaptive within the context of talent development (Flett and Hewitt, 2005; Stoeber, 2011; Hill et al., 2015; MacNamara and Collins, 2015), have also been observed as a precursor to major depression, anxiety disorders and eating disorders (Sassaroli et al., 2008), with such influence tending to center around evaluative concerns (DiBartolo et al., 2007). As well as the associated maladaptive aspects of perfectionism, eating disorders such as anorexia nervosa and bulimia nervosa also bring with them other issues, such as potential nutrient deficiency, further compromising the physical adaptation to exercise and initiating other cycles of underachievement-depression. Indeed, although often perceived as a predominantly female issue—perhaps in part due to the female athlete triad (Nattiv et al., 2007) and the lack of a male equivalent (Thompson and Sherman, 2014)—both male and female athletes are recognized as at risk of disordered eating and eating disorders (Baum, 2006; Thompson and Sherman, 2010). Furthermore, some sports carry a greater risk of eating disorder, such as aesthetic sports (e.g., gymnastics), sports where low body fat is advantageous (e.g., long distance running), weight-making sports (e.g., boxing) (Baum, 2006), and sports such as rugby, where increased body mass is advantageous (Hill et al., 2015; Till et al., 2015). As such, issues surrounding body image and related eating disorders are pervasive throughout sport (Kong and Harris, 2015). It may also be that the stage of development and age of the athlete represent even more important considerations than the sport itself. Indeed, Thompson and Sherman (2014) have identified young developing athletes as a particularly high risk group, citing less available support, lower levels of awareness and being at a high-risk age as extra risk factors. Unfortunately, there is relatively little evidence to support this and further research is warranted with this at-risk cohort. So, despite coaches acknowledging the impact of mental health issues in TID and given that these coaches are often the primary identification tool for such issues (Sherman et al., 2005), they report a lack of understanding about clinical mental health issues in sport (Hill et al., 2015). With this in mind, the purpose of this study was three-fold. The first aim was to identify the range of mental health issues that may impact on such individuals, both as developing athletes and as adolescents, along with potential consequences for the development process. The second aim was to investigate specific risk and protective factors that may be associated with, or incorporated into talent development environments. Finally, this study sought to examine current practices around identification of mental health issues within a TID setting, with a view to addressing potential inefficiencies.

## Methods

### Participants

This study set out to investigate the range of clinical mental health issues that impact upon developing athletes and high achieving adolescents through a series of cross-sectional, retrospective qualitative interviews. Such an approach has been widely adopted throughout sport psychology literature (e.g., MacNamara et al., 2010) as a way of identifying phenomena and eliciting high levels of information-rich data, whilst acknowledging limitations relating to truthfulness and self-report bias (Patton, 2002; Amis, 2005; Atkinson and Delamont, 2005). A purposive, criterion-based sampling approach was adopted (Patton, 2002), with potential participants identified based on their clinical qualifications, roles and experience of working with developing adolescents. Semi-structured interviews were conducted with clinicians specializing in children and young people and/or athletes (*n* = 8; 2 male, 6 female), in a bid to draw on their unique understanding of issues that may impact upon developing athletes and high achieving adolescents. The participants' experience ranged from 13 to 31 years of providing clinical support (*M* = 20.2 years, *SD* = 7.91), with all participants experienced in working with adolescents, and six participants experienced in both sport and adolescent environments.

### Procedure

Ethical approval was obtained from the authors' institutional ethics committee prior to the commencement of the study, with informed consent obtained from all participants and with confidentiality assured. A semi-structured interview guide was developed (see Supplementary Material), designed to explore the different types of clinical issues experienced and their consequences, along with follow-up probes and prompts to elicit data in specific areas of interest. Topics addressed included the types of issues and their impact (e.g., “Based on your experience, can you describe the types of issues that have been presented in developing athletes?”), the role of the environment (e.g., “What protective factors do they offer?”), and issues surrounding identification and assessment (e.g., “What observable behaviors might give you cause for concern in a developing athlete?”). Interviews were conducted by the first author, who had previous experience in interviewing, qualitative methods and talent development. Interviews lasted between 45 and 76 min, (*M* = 60.3 min, *SD* = 11.01 min), preceded by a briefing and an introduction, and were conducted at locations chosen by the participants.

### Data analysis

Interviews were transcribed verbatim, with the researcher's notes, questions and annotations regarding possible misinterpretations added. These were then returned for participant checking, allowing the participants opportunity to clarify meanings in a bid to enhance credibility (Patton, 2002; Amis, 2005). Clarifications were received from two practitioners, with the appropriate amendments made to the transcript prior to analysis. In the first instance, content analysis was undertaken in line with the recommendations of Côté et al. (1993), whereby meaning units were created from raw data segments. Inductive content analysis was then performed, whereby meaning units were grouped together in emergent categories based on their similarity to each other and distinction from other categories (Côté et al., 1993; Patton, 2002). This process was then repeated in order to generate higher-order themes until theoretical saturation was reached, whereby all new meaning units fit into the existing code structure (Strauss and Corbin, 1998).

As the researcher is the primary data collection tool within qualitative interviewing, the scope for researcher bias must be recognized. In a bid to aid credibility, conformability and dependability (Strauss and Corbin, 1998; Patton, 2002; Amis, 2005), an independent researcher experienced in both qualitative methods and talent development was invited to critically analyze the emergent categories to ensure they accurately reflected the participants' quotations. Where this resulted in disagreement between the researchers, interpretations were put forward until an agreed explanation was found (as per Patton, 2002), leading to the re-categorization of four items.

## Results

The purpose of this study was to identify the range of clinical issues experienced by clinical psychologists in a talent development setting. Four main over-arching themes were identified in the study: behavioral indicators; associated risk factors; associated protective factors; and identification and diagnosis issues. An overview of the emergent themes is also presented in Table 1, with the themes italicized within the main text to aid clarity.

**Table 1:** **Clinical issues in talent development**.

**Higher order theme**	**Theme**	**Sub-theme**
Behavioral indicators	Anger and aggression	–
	Anxiety and OCD-type behaviors	Obsessive compulsive disorder
		Performance anxiety
		Social anxiety
		Superstition
	Changes in behavior	–
	Communication and interaction	–
	Depression and low mood	Rumination
		Withdrawal
	Disruptive behavior	–
	Eating disorders	Excessive focus on bodyweight
		Hiding the body
		Low energy
		Weight loss
	Emotional suppression	–
	Injury and illness behavior	–
	Non-typical development patterns	–
	Not adhering to coaching and authority	–
	Obsession and perfectionism	–
	Phobias	–
	Self-medication	–
	Self-harm	–
	Sleeplessness	–
Identification and diagnosis issues	Assessment and screening tools	–
	Awareness of issues and symptoms	–
	Individualized approach	–
	Multiple causes of symptoms	–
	Need for clinical skills	–
	Non-disclosure by athletes	Lack of awareness
		Reluctance to disclose
	Normal developmental behavior	**–**
	Observation	–
	Understanding the athlete's environment	–
Protective factors	Open and supportive coaching environment	Communication
		Safe environment
		Structure and purpose
	Social protective factors	Interested role models
		Parental and family support
		Peer support
		Effective relationships
Risk factors	Body image	–
	Developmental risk factors	Adolescence
		Attachment and identity
		Cognitive ability
	Family and home environment	Pushy parents
	Performance environment	Club culture
		Competitiveness
		Deselection, transition, and exit
		Excessive downtime
		Performance pressure
	Social factors	Isolation and removal from peer group
		Peer competition
		Peer pressure
		Social evaluation
	Unbalanced approach to sport	–

### Behavioral indicators

A host of behavioral indicators were identified as being indicative of, or a precursor to, mental health issues. The primary indicator identified by all of the participants was that of *changes in behavior and/or performance*. Deviations away from an individual's regular behavior—particularly those that were currently unexplained—were highlighted as fundamental, and typified by the following example:

Change. Identifying change is key. It's really a shift, and it's a shift over a period of time. So it's not just a one off, but if you get persistent behavioral change, then I would say that's a very important feature (Clinician 1)

Given its generality, such a behavioral indicator places an emphasis on the need to be familiar with an individual's regular patterns of behavior, a point also reflected in the data. (e.g., “And if they know the kids really well, some [coaches] are good at picking up [the changes], if children aren't their normal self.” (Clinician 4). *Disruptive behaviors* were also identified as potential indicators, along with issues around *not adhering to coaching and authority, displays of anger and aggression*, although these were not symptomatic of a particular clinical issue; rather, they were recognized as more general “warning signs” that warrant further investigation: “…[you find out] more when you talk to them. So it would be more around the clinical questioning, I suppose, and trying to get underneath when things aren't working well” (Clinician 7).

Along with these more general characteristics, a range of behavioral issues associated with specific clinical issues were also identified. Indicators associated with *eating disorders* included unexplained or unscheduled weight loss [e.g., “And it's looking for the usual thing—a kid getting skinnier, without having suddenly put on a growth spurt” (Clinician 3)], low energy levels [e.g., “The heavy load sports—swimmers for example—couldn't keep going at a heavy session. And coaches have noticed that's been a lack of stamina has come up” (Clinician 4)], hiding the body with excessively baggy clothes, feeling the cold more readily than normal (or than their peers), and restricted eating, as typified by this example:

“I can think of a top climber who was eating mackerel salads for weeks, and I mean just a piece of lettuce and a piece of mackerel for tea; really small amounts to lose as much as possible before trying an ascent of a hard route” (Clinician 7)

The potential influence of weight on performance was cited as a key contributing factor, with practitioners acknowledging the delicate balance:

It's about getting that balance just right…when you get a performance benefit from losing a bit of weight, it can be quite appealing to keep going with it” (Clinician 6)

The extent to which this could manifest itself was demonstrated by data from Clinician 4, with athletes taking seemingly drastic measures:

I think with things like weight-making sports, you've got to get in and address it early. There was someone at the [city removed] Olympics in [name of sport] who cut their hair to try to make weight. Now the amount of hair [they] removed wasn't going to make a difference in the slightest. (Clinician 4)

Indicators of *anxiety* were reported throughout the data and were recognized as the most common types of issue presented to the clinical sports practitioners; even amongst the non-sport clinical psychologists, anxiety was reported as commonplace:

So the major problems that we see come through [name of organization], a lot of that is around anxiety (Clinician 3)

In terms of my clinical experience, I would say anxiety is more prevalent than depression, certainly more prevalent than psychosis, but we do get a skewed view in terms of children coming to see us.” (Clinician 1)

Certainly within a sporting context, *performance anxiety* was reported as a contributing factor [e.g., “You'll certainly come across a lot of people who are very, very anxious before games. They're not sure how to channel that anxiety or those symptoms.” (Clinician 3); “…and having to manage anxiety around performance is important” (Clinician 1)]. Along with the performance aspects, issues around *social anxiety* were also prevalent [e.g., “…a young person I worked with as well had massive social anxiety, as in could barely even talk to me” (Clinician 7)]. A range of performance-based consequences were attributed to or influenced by these anxieties, including panic attacks, communication breakdown, poor decision making, nervousness, the “yips” and lost move syndrome.

Sharing a high level of comorbidity with anxiety disorders (American Psychiatric Association, 2013), *obsessive compulsive-type behaviors* were prevalent throughout the data, and were employed by people in a bid to control their environment [e.g., “…actually it's about controlling their world. It's not just anxiety, it's controlling their world that feels out of control, even though maybe it isn't, and it's just one tiny aspect of it.” (Clinician 2)]. This manifested itself through a range of behaviors such as checking and rituals [e.g., “It's noticing things like do they have a ritual when they're packing their bags?…I think towels were always lined up for [name of athlete]—I think it's noticing things like that” (Clinician 4)]. Similarly, *superstitions*—differing from OCD-type rituals in their unreasonable beliefs around cause and effect, rather than a compulsion to act upon intrusive thoughts (Živanović et al., 2012; American Psychiatric Association, 2013)—were also recognized to impact upon an individual's performance and anxieties:

A lot of superstitious behavior is around in sport, and I think it's getting people to recognize that and then taking action. “This is a superstition, it's not a fact”…It's picking up things like that, that maybe gets “Oh I can't do this, I've not got my lucky rabbit's foot with me” (Clinician 4)

*Depression*, also highly comorbid with anxiety, was identified as a key issue, with behavioral indicators such as persistent *low mood, rumination* [e.g., “…ruminating on mistakes and getting very stuck in that ‘I have failed”—it's all black and white. They're actually stuck in their heads.” (Clinician 7)], *withdrawal* [e.g., “Are they not turning up? Are they ill a lot?” (Clinician 2); “They become quite isolated within the environment” (Clinician 5)], and *sleeplessness* [e.g., “But in terms of the younger people that I work with it's been sort of not sleeping, going back to ruminating” (Clinician 8)]. *Sleeplessness* was also associated with *anxiety*, and was a particular issue when away from home or at training camps, as highlighted in the following example: “They were perhaps struggling with sleeping when they were away from home, things like that, and obviously [the coaches] didn't want to give them sleeping tablets, so teaching the behavioral techniques to manage anxiety [was important]” (Clinician 4). As a precursor to *depression, emotional suppression* was recognized as potentially having drastic consequences to an athlete's development, as highlighted in the following example:

So things trundle along and then all of a sudden you get burnout…Fundamentally, that suppression, that avoidance, that lack of acknowledgement of the emotional impact of what they're doing, longer term can set up high risk for depression—that bottle-bang…When a kid, all of a sudden one day turns around and says I don't want to do it anymore. (Clinician 5)

*Obsession and perfectionism* were recognized as a common feature, particularly amongst the clinical sport psychologists when compared to their non-sport counterparts. This was characterized by extreme perspectives and “binary” thinking [e.g., “I think you also have a range of what might be called extreme perspectives, because players will talk about that they need to be unbelievably focused so they'll be successful. (Clinician 3); “It tends to be very much about the black and white thinking, that kind of all or nothing stuff. So either I've done this perfectly or I've completely failed” (Clinician 7)].

Issues around *self-harming* and around *self-medication* were recognized as features of the general clinical population, but were not reported by the participants as prevalent within sport. However, due to the qualitative nature of this study, and in particular the use of few, high quality subjects, caution should be taken when drawing any quantitative conclusions; an absence in this study does not necessarily suggest that this is not an issue in a sporting context.

### Risk factors

Of the risk factors identified, *family background and home life* was the most widely acknowledged. An *unstable home life* was cited as a key issue (e.g., “If it's an unstable home, if there's trauma in the person's background, then they don't have the resources themselves, the resilience to deal with [setbacks]” (Clinician 2); “…Often dating back to divorce; the parents separating, and just having a hard time at home.” (Clinician 4). Similarly, other family-related issues have been seen to have an impact, such as caring for a parent:

So kids who have become carers in any form, in my view always have a certain amount of struggle as to their role in life, as to whether they take care of people or whether they take care of themselves, and their childhood is compromised. (Clinician 2)

However, a stable family background does not in itself mitigate any associated risk, as each of the clinical psychologists highlighted the potentially detrimental role of *pushy parents*:

One of the kids that I was thinking about who came to me very, very socially anxious, was more pushed in to the coaching by his dad than he wanted to be himself…he was a very talented climber and I think he's pretty much off the radar now. (Clinician 7)

But there could be a lot of pressure. I'll never forget in skating, sitting at a competition and people going on about “their parents must be so embarrassed,” and I was thinking that's really interesting, the comments and the investment that the parents are making (Clinician 4)

The *performance environment* was recognized to bring with it a range of factors that could increase the risk of developing mental health issues. With wide-ranging consequences (or at least perceived consequences) surrounding performance failure, *pressure to perform* was a key driver for many of the potential associated issues (e.g., “everything is task oriented [i.e., tasks must be completed], goal driven—that, we know, or at least we have strong indicators that that style over time increases our risk of mental health problems” (Clinician 6). The *competitive nature* of the environment was particularly associated with *hiding weakness*, which was seen to carry potential negative consequences:

And if that mentality of remaining tough and not wanting to show any weakness on the pitch, if you take that in to your daily life, the potential is you can't show any sort of weakness whatsoever, and that will stop you getting help and support. (Clinician 3)

Such a competitive environment and the associated impression management was also viewed as being potentially self-perpetuating:

Sometimes the things that are valued in elite sport environments are the very things in the short term that look really good, but in the long term increase the risk of future difficulties…So you hear things like mature for their age, independent, driven, focused. And of course it's not just the individual—the system gets seduced to reinforce that, as do the coaches. So you've got an individual who's perfect, who's the ideal kid—they worry the hell out of me. (Clinician 5)

As a fundamental part of the talent development pathway, *transitions, deselection and exit* were identified as potential obstacles that, without the appropriate skills and/or support, could increase the risk of a young athlete developing mental health issues:

Well the obvious one is not making it, and then what does that mean for your life? If you look at a CV and everything on it is going toward one goal, and they don't make that goal, where do they go then? (Clinician 1)

If there's a transition—I'm a 17 year-old, I'm idolized by everybody and then I'm put up in to the 19s or in to the senior squad, and I'm now not the best in the class—how will they cope? (Clinician 5)

As “micro-transitions” themselves, periods of *injury* also posed potential risk [e.g., “Even if you're out for six weeks, it's a big issue because you don't feel part of that training squad, that camaraderie, you become distanced from it. Watching from the side lines is a lonely place” (Clinician 3)], especially if rehab is problematic or is over a long period of time:

I think we mustn't forget as well that with chronic long term injury, people can get depressed too, just because they're not getting that—and that was often a factor with people in their rehab—they weren't getting where they wanted to be. (Clinician 4)

Away from the performance environment, *developmental risk factors* included *adolescence* itself [e.g., “I think in adolescence, and understanding the nature of adolescence, which is very black and white, I think it's only as we get older that we realize that life has more gray.” (Clinician 2)]. Differing levels of physical maturity was seen to be potentially problematic both on an individual level for the young person and how it affects their relationships with others:

Adolescence is such a time when you're super sensitive as to the world around you, so it's a very insular thing, but it's also about how I fit in the world, and if you're not fitting in for any tiny thing then it seems to exacerbate everything else. (Clinician 1)

Children are not mini adults and we treat them as mini adults. They're developing, so physically they may develop, a 15 year-old in rugby, for example, they may physically look like they're men but they're still sometimes little boys. (Clinician 5)

Similarly, issues around *identity and attachment* were shown to have developed over time, with an attachment to the sport often viewed by the individual as a valuation of their own worth. This can then become problematic if their sport performance subsequently dips:

So being good at something gives you a sense that you're a good person, therefore if you start to play up and you're not so good at something, and this can work one way or another, then the very thing that keeps you thinking you're a good person, you're not doing so well, and that can tip you in to a negative spiral. (Clinician 5)

*Social risk factors* included issues around *peer pressure* [e.g., “What are your peer group doing? Are you going to do the same or are you aware that your sport needs to be your focus?” (Clinician 8)], *social evaluation* (e.g., “[Sport]'s a really small community, so everybody knows each other and there's that sense of your performance is always being evaluated by somebody else. So that's one of the biggest things that I think holds people back” (Clinician 7), and due to the unique nature of talent development environments, *peer competition*, whereby your peers within a system are also your rivals for the finite number of positions available at elite level. This was seen to compromise the effectiveness of peer support.

If your social network is around those squads of 20–30 players, there'll always be jealousy. Players will think I should have got that contract, so that will impact on the potential social interactions with those people again for the future. (Clinician 3)

Often it's when they've become very much attached to their peer group, and that isn't very supportive particularly, and again it's not a place for them to necessarily talk about things that are going wrong for them, because they might see that as showing weakness (Clinician 1)

### Protective factors

In contrast to the risk factors identified above, a range of protective factors were also identified. Of primary importance were the *social protective factors*, deemed to have a significant positive influence on an adolescent's development. Of these social factors, the role of *parental and family support* was viewed by the participants as fundamental to wellbeing throughout development:

There's something out of child development that says there are some kids that have got immune to certain things because they've had good supportive upbringings, so although they might be upset by a bereavement or a separation of their parents for example, they might not be as bad as others because there may be a good stable grounding behind them. (Clinician 1)

Similarly, having an *interested role model* to look up to was recognized to have a positive effect throughout development, again through providing stability [e.g., “Sometimes being almost like a surrogate parent—the stable person in their lives, being there for them no matter how much they're acting out. That you're still there but you're not tolerating necessarily” (Clinician 2)]. Despite the nature of many talent development environments necessitating between-peer competition (e.g., for professional contracts), *peer support* was evident as a protective factor in some circumstances (e.g., “A lot of players will find support, so if a couple of players are injured, if they're doing the same rehab at the same time, [they'll help each other through]” (Clinician 3)). Such utilization of social support was recognized to be underpinned by the ability to form good *relationships*:

It wasn't about ability, so it wasn't the brightest from there that did best, it was the ones that did best in other areas so that sort of being able to create and make good relationships seems to be a very key element. (Clinician 1)

An *open and supportive coaching environment* was seen as a valuable way to encourage building those types of relationships, as well as providing opportunities for role modeling:

But from a coach's perspective, it's about opening up a conversation, if it's possible to do so. You have to have an environment to do that…. If it isn't with the coach, who's it going to be with? You have to have that link person or somebody who's trusted enough to speak to. I think trust and confidentiality is the key really. (Clinician 8)

### Identification and diagnosis issues

Throughout the data, key issues around identification and diagnosis were raised. Of these issues, all participants highlighted the need for greater *awareness of clinical issues* that impact upon adolescents. This requirement was not limited to the coaching environment, but was felt to be an issue for everybody deemed part of the young person's life. This increased awareness was not only deemed important to help identify the issues more effectively, but also to increase awareness around how to take the first steps in addressing the issue, as highlighted by the following example:

I think awareness is really important. I was just speaking to a father the other day about his child and he just didn't have a clue. He's obviously a very nice man, but he didn't have a clue about how you got help, what help was there, and he was a very able individual. It wasn't like he was somebody who didn't know life, but when he was faced with anxiety in his child, he didn't have a clue what to do. (Clinician 1)

However, simply increasing the awareness of symptoms was recognized as problematic, due to the *multiple causes of symptoms*, especially symptoms associated with normal adolescent development [e.g., “Especially dealing with teenagers. They've got a hell of a lot on their plate, haven't they, so you can't be sure what was causing the issue.” (Clinician 7); “So sometimes it can be a little difficult easing out what's normal adolescent behavior and what actually we should be worried about.” (Clinician 4)].

Muddying the waters further is the issue of non-disclosure. *Non-disclosure by athletes* was attributed to two main factors: a lack of self-awareness and a reluctance to disclose their concerns about their mental wellbeing. *Lack of awareness* was particularly an issue for the younger adolescents, with some issues more likely to be picked up than others [e.g., “Insight's a difficult thing, and sometimes I think it is hard to know what's wrong because you're just feeling rubbish. Or if things aren't going right or nothing seems to be right at the moment.” (Clinician 1); “Whereas they might actually say “Oh, I do feel funny” and they might be experiencing a panic attack. They're more likely to talk about that than a feeling of sadness.” (Clinician 4)]. A *reluctance to disclose* to somebody was recognized to occur for multiple reasons. Inhibiting factors included that of stigma around mental health issues [e.g., “I think across many sports I think stigma is a really big issue” (Clinician 3)], the potential impact it may have on future selection [e.g., “…you may be worried about the potential impact—it depends upon the coach, I think” (Clinician 8)], and fear of upsetting others, particularly parents [e.g., “The issue that I've found, …is that children and young people really don't like telling their mum and dad because they don't want to upset them.” (Clinician 2)]. Away from the individual, *non-disclosure by others* was also recognized as a significant barrier. Despite recognizing potential issues in adolescents, significant others were often seen to attribute them to developmental “phases” and were therefore unlikely to seek further help in addressing them:

And that notion of “it's just a phase,” generally speaking, probably isn't a great thing. It can be, you know, it can be at times, but if something persists, then you do need to go about getting help…I think parents try to be very optimistic. They don't really like the idea of their child not being quite right. (Clinician 1)

In order to address this obfuscation, a range of actions were identified as necessary. *Observation* was utilized on an individualized level, in order to pick up on any potential issues [e.g., “When you've got them there at an academy, you're going to have at least one coach who would pick up perhaps some of the issues as well.” (Clinician 4); “I think it's about extremely observant people, and it comes back to people getting to know each young person as best they can, so that actually that's when you start to notice when things are different.” (Clinician 2)], clinical questioning skills were employed by practitioners where appropriate, and a range of *assessment and screening tools* were administered, including the Generalized Anxiety Disorder 7 item scale (GAD-7; Spitzer et al., 2006) and the Patient Health Questionnaire 9 item scale (PHQ-9; Kroenke et al., 2001):

In the education sessions we use with players, we use the PHQ9 and GAD7. We don't ask the players directly, we ask them to think about people who they might know who might be stressed, which is usually coaches. So they get to listen to those ideas around that, or perhaps assess a former player and get them to tell us if they think there's a problem or not. (Clinician 3)

However, there were several notable limitations to such an approach, including the emotional literacy of the subject [e.g., “So I think for some of the people, there's a degree of emotional literacy that you need first before you could get anywhere with even a questionnaire.” (Clinician 7)], and the sensitivity of the assessment tool itself [e.g., “You have to be more subtle, which is why questionnaires and these things fundamentally don't work, because you don't pick up” (Clinician 6)]. In recognition of such limitations, assessment tools were used by practitioners as part of a triangulation process as part of an assessments, and sometimes as a guide for more informal conversations.

## Discussion

A range of clinical issues were identified within the data with negative consequences for young developing athletes, including eating disorders, anxiety, and depression. Due to the qualitative and exploratory nature of this study, the results do not indicate an order of prevalence or importance of issues; only their existence in the specified domain. However, the consequences of such issues, if not diagnosed and/or managed appropriately, were recognized to increase the likelihood of derailment from the talent pathway. Accordingly, there is a clear need for effective identification and intervention strategies in order to ensure the wellbeing of the athlete and maintain the efficacy of the talent development process. Such issues had clear and specific behavioral indicators specific to the illness that trained clinicians could readily identify. Concurrently, the onset of mental health issues in young people was also reported to yield a more general set of indicators in the form of behavioral change. It was suggested that these more general “warning signs” were readily identifiable by those without a clinical background, on the proviso that they were familiar with the individual, their circumstances and their normal patterns of behavior; a fact supported by existing research (see Hill et al., 2015; Mazzer and Rickwood, 2015).

However, given that athletes often have close working relationships with their coaches (Sherman et al., 2005; Davis and Jowett, 2014), yet the participants in this study raise the point that many young people still “slip through the net,” there becomes apparent the need to identify and address this discrepancy; both for the benefit of the talent development system, and more importantly, for the welfare of the developing athletes. Given the logistical unfeasibility of providing coaches with a comprehensive clinical skillset, in order to address this issue, a more systemic approach is needed.

As the data suggests coaches who have long-standing relationships with their athletes will notice the behavioral change, interventions designed to optimize the coach-athlete relationship, along with education around changes in behavior and understanding the associated implications are recommended, in order to address the issue of non-disclosure by others. As part of a triangulation process, similar to that used by the participants, ecologically validated assessment tools could aid in bringing to the fore specific “warning signs” that may otherwise have gone unnoticed. Further education around signposting, enabling the coaches to know what to do next—and more specifically—who to contact, is vital, if effective intervention and management of any potential clinical issue is to occur. As a more holistic approach to identifying clinical issues in talent development, the incorporation of trained clinicians in to talent development environments would potentially be an effective solution to both identification issues and that of appropriate clinical intervention.

Despite such recommendations for improving the effectiveness of identification and intervention around clinical issues in talent development, the key point of how to limit the development of such clinical issues still remains. In order to attempt to address this, examination of the associated risk factors and protective factors is required. Of the risk factors identified within the data, social issues around an athlete's background and family life were deemed the most impactful by the participants. The role of the family was seen as particularly important, given the psychological stress caused by traumatic life events such as bereavement (Sarkar et al., 2014), parental divorce (Amato and Keith, 1991; Amato and Sobolewski, 2001), and caring for family members (Aldridge and Becker, 1999). Such key events, along with the identified issues readily associated with adolescence (MacLeod and Brownlie, 2014), are not confined to the domain of talent development, but are more general in both nature and genesis—a fact borne out by the data. As such, there is limited practical scope for preventative or remedial action by the talent development system to mitigate the impact of these issues, other than by helping to support (directly or indirectly) the young athlete through the process. However, one family-based risk factor where the talent development environment can have a pro-active, positive impact is in mediating the maladaptive influence of parental behavior, in particular the role of the “pushy” or “problem” parent.

As key stakeholders in the talent development process (Pankhurst et al., 2013), parents are highly influential in establishing an athlete's motivational climate through their values and behaviors (Gould et al., 2008; Gustafsson et al., 2015). Consequently, athletes are not only able to benefit from supportive parents, but are also susceptible to a parent's own anxieties around their child's performance (Beidel and Turner, 1997; Ginsburg, 2009). Given the amount of time, money and emotion invested by parents in their child's sporting success, it is perhaps then unsurprising that such anxieties can manifest themselves as behaviors detrimental to the athlete's wellbeing and development, such as over-involvement (Wuerth et al., 2004), negative verbal behaviors during performance (Kidman et al., 1999), and negative debriefing (Elliott and Drummond, 2015). Despite the likely underpinning good intent, there appears to be a lack of common understanding of the parental role between parent and child in a talent development setting (Kanters et al., 2008), and it is the perceptions and possible misinterpretations of these behaviors that, in turn, often act as sources of acute stress for the developing athlete (Babkes and Weiss, 1999; Puente-Díaz and Anshel, 2005; Kanters et al., 2008). Worryingly, issues such as anxiety and fear of failure having been shown to transfer from parent to child, however such transference has also been demonstrated as amenable to intervention (Sagar and Lavallee, 2010; Ginsburg et al., 2015). Similarly, group education-based interventions have also proved effective at facilitating adaptive parental support through the provision of “real-world” strategies and improved awareness (e.g., Richards and Winter, 2013). Based on the apparent success of such programs and given the established need within a talent development setting, we would propose proactive, education-style interventions aimed at promoting parental awareness of the issues around talent development, and in particular parental behaviors and their potential impact upon their child's mental well-being.

Given that the role of the talent development environment is to prepare a developing athlete for elite level competition, and that elite level sport is widely recognized as high pressured and highly competitive (Pensgaard and Roberts, 2000; Jordet, 2009), addressing the risk to mental wellbeing associated with the environment's competitive nature proves problematic, and must be done with care. A reduction in the level of competitiveness and/or pressure within a talent development environment may—in the short-term—allay any concerns over a developing athlete's mental health, yet in the long-term may only serve to under-prepare the individual for what lies ahead; thus potentially exposing them to the risk of potential mental health issues in the future. As such, we would propose a three-stage strategy in addressing this issue. First, careful consideration must be given to the potential impact on an individual's mental wellbeing of any likely outcome, as part of a professional judgment and decision making process (see Martindale and Collins, 2005, 2013). This would require a good level of awareness of the individual, the environment, and of mental health issues, and would therefore need to be underpinned by specific training where appropriate. For example, a transition from an academy program into elite competition brings with it many pressures, such as a heightened emphasis on results and increased expectation. Such pressures are often associated with fear of failure (Sagar et al., 2007), and the subsequent defensive behaviors (e.g., avoidance; Birney et al., 1969) are associated with mental health issues (Grant et al., 2013). Accordingly, appropriate measures to mitigate the detrimental impact of such pressures may be required. Second, regular monitoring of both coping skills (e.g., psychological characteristics of developing excellence; MacNamara et al., 2010) and of mental wellbeing would be required, in order to maintain the appropriate level of challenge for the individual and to target the necessary areas for development. Finally, appropriate support and signposting must be provided where necessary, in order to identify and address any potential mental health issue as soon as possible, with timely intervention often the key to successful treatment (Kamm, 2008).

In many respects, the range of associated protective factors offer a reflection of the risk factors discussed earlier. For example, whilst the role of the family, the competitive nature of the talent development environment, and social evaluation from peers have all been identified as sources of stress, family and parental support, an open and supportive coaching environment, and peer support were all said to play a significant role in protecting a developing athlete's mental wellbeing. The fact both the family environment and the talent development environment can offer both protection from and susceptibility to mental health issues highlights the importance of the effectiveness of the relationships formed between the people within these environments; a point borne out by the study's data. As such, talent development environments should seek to establish and actively promote such relationships throughout their system. Concurrently, supportive family relationships must also be fostered wherever possible, in a bid to offer each young athlete the best possible protection and support.

## Conclusion

The aim of this study was to address the nature of clinical issues within talent development, and their associated issues. Following a series of qualitative interviews with clinical practitioners with experience of working with adolescents and/or elite developing athletes, four key areas were identified: behavioral indicators; associated risk factors; associated protective factors; and identification and diagnosis issues. In a bid to address these issues, several key recommendations were made. First, the incorporation of clinical expertise into the talent development process is crucial (either through direct employment or referral), as without such expertise, diagnosis and intervention cannot occur. Second, as those best placed to identify more general warning signs of mental health issues, coaches and support staff are likely to require training and support in dealing with such issues. Thirdly, as part of a triangulation process, an ecologically validated and reliable assessment tool would aid in the regular monitoring of athletes' coping skills and mental wellbeing throughout the development process. Finally, as effective relationships are fundamental to an environment's protective qualities, such supportive relationships need to be established and actively promoted throughout. Through the implementation of such measures, the effectiveness of talent development processes will be improved due to the potential decrease in talent derailment. This is, however, of less significance than the positive impact it will have on the mental wellbeing of young athletes.

## Author contributions

All the authors have made substantive contributions to the article and assume full responsibility for its content. AH, DC, AM, SR designed the study, developed the methodology, and wrote the manuscript. AH collected the data and AH, DC, and AM conducted the data analysis.

### Conflict of interest statement

The authors declare that the research was conducted in the absence of any commercial or financial relationships that could be construed as a potential conflict of interest. The reviewer and handling Editor declared their shared affiliation, and the handling Editor states that the process nevertheless met the standards of a fair and objective review
