# Supplementary material for: Examining the Role of Mental Health and Clinical Issues within Talent Development
Source: Front Psychol. 2016 Jan 11;6:2042. doi: 10.3389/fpsyg.2015.02042 (PMC4707871; doi:10.3389/fpsyg.2015.02042)
Supplement: Supplementary file 1 [file DataSheet1.DOCX]

Supplementary Material

Examining the Role of Mental Health and Clinical Issues within Talent Development

Andy Hill*, Áine MacNamara, Dave Collins, Sheelagh Rodgers

*** Correspondence:** Corresponding Author: ahill@uclan.ac.uk

# Supplementary Data

Section 1: Description of apparent clinical issues in the TD setting

Based on your experience, can you describe the types of issues that have been presented in developing athletes?

- What symptoms/presentations are associated with these issues?
- Are some more prevalent than others? Is there a reason for this?
- Can you describe an example of how these issues impact upon the development process and the athlete’s day-to-day life, both in and out of sport?
- What are the consequences of not addressing these issues?
- Are these issues manageable over an athlete’s development?
- Are some of these issues likely to lead to the premature derailment of an individual’s sporting career? If so, how?

Section 2: Risk factors and protective factors associated with talent development and high-achievers

In your experience, does sport, and in particular talent development, bring with it any particular inherent risk factors that may make a developing athlete more susceptible to clinical issues?

- Can you describe examples of these inherent risk factors?
- Does the ‘system’ play a role, either positively or negatively? If so, How?
- How might these risk factors and their subsequent associated issues impact upon both an individual’s mental health and their development as an athlete?

Do talent development environments offer a protective element to high achieving adolescents?

- What protective factors do they offer?
- Can you describe examples of this in action?
- Are these factors only available through TDEs or also through other ways?

In your experience, what types of mental health issues tend to be associated with high achieving adolescents, in particular within a sporting environment?

- Can you describe examples of where these have impacted upon an athlete’s development, either personally or professionally?
- In your opinion, are there steps that can be taken by clubs, academies and other talent development environments to mediate any such issues?

Section 3: Identification of potential clinical issues within an applied setting

What observable behaviors might give you cause for concern in a developing athlete? How would you screen for it?

- What characteristics would make an individual stand out within a TDE as potentially requiring support?
- Do these behaviors have multiple causes?
- Would such behaviors be easily observable by untrained individuals or those with a limited understanding of clinical issues?
- Is there a role for assessment tools such as the PHQ-9 or GAD-7? Are they useful in a talent development setting?
- Are other tools available and/or used?

How does the referral process work? Do clients approach you directly or are they referred by, for example, club doctors, coaches, GPs, parents, etc.?

- Is this process effective?
- Do you feel that athletes may not be being identified as needing specialist help? If so, how are they falling through the net?
- How could this process be improved to address these issues?

Is there any further information you’d like to add on any of the previous sections, or a question that would be beneficial but I haven’t thought to ask?
